# Rectus Abdominis Flap Replantation after 18 h Hypothermic Extracorporeal Perfusion—A Porcine Model

**DOI:** 10.3390/jcm10173858

**Published:** 2021-08-27

**Authors:** Anne Sophie Kruit, Dominique van Midden, Marie-Claire Schreinemachers, Erik Koers, Her Zegers, Benno Kusters, Stefan Hummelink, Dietmar J. O. Ulrich

**Affiliations:** 1 Department of Plastic and Reconstructive Surgery, Radboud University Medical Center, 6525 GA Nijmegen, The Netherlands; mcschreinemachers@yahoo.com (M.-C.S.); Stefan.hummelink@radboudumc.nl (S.H.); dietmar.ulrich@radboudumc.nl (D.J.O.U.); 2 Department of Pathology, Radboud University Medical Center, 6525 GA Nijmegen, The Netherlands; dominique.vanmidden@radboudumc.nl (D.v.M.); Benno.Kusters@radboudumc.nl (B.K.); 3 Department of Cardiothoracic Surgery, Radboud University Medical Center, 6525 GA Nijmegen, The Netherlands; Erik.koers@radboudumc.nl (E.K.); her.zegers@radboudumc.nl (H.Z.)

**Keywords:** VCA, ex vivo preservation, composite tissue transplantation, mid-thermic storage, free flap

## Abstract

Cold storage remains the clinical standard for composite tissue preservation but is time-limited. A long ischemia time during surgery will adversely affect postoperative outcomes due to ischemia-reperfusion injury. Extracorporeal perfusion (ECP) seems to be a promising alternative for prolonged preservation, but more evidence is needed to support its use and to identify optimal perfusion fluids. This article assessed musculocutaneous flap vitality after prolonged ECP and compared outcomes after replantation to short static cold storage (SCS). Unilateral musculocutaneous rectus abdominis flaps were raised from 15 pigs and preserved by 4 h SCS (*n* = 5), 18 h mid-thermic ECP with Histidine–Tryptophan–Ketoglutarate (HTK, *n* = 5) or University of Wisconsin solution (UW, *n* = 5). Flaps were replanted and observed for 12 h. Skeletal muscle histology was assessed (score 0–12; high scores equal more damage), blood and perfusate samples were collected and weight was recorded as a marker for oedema. Mean histological scores were 4.0 after HTK preservation, 5.6 after UW perfusion and 5.0 after SCS (*p* = 0.366). Creatinine kinase (CK) was higher after ECP compared to SCS (*p* < 0.001). No weight increase was observed during UW perfusion, but increased 56% during HTK perfusion. Following 12 h reperfusion, mean weight gain reduced 39% in the HTK group and increased 24% in the UW group and 17% in the SCS group. To conclude, skeletal muscle seemed well preserved after 18 h ECP with HTK or UW perfusion, with comparable histological results to 4 h SCS upon short reperfusion. The high oedema rate during HTK perfusion remains a challenge that needs to be further addressed.

## 1. Introduction

Skeletal muscle is one of the most vulnerable tissues to ischemia, with a maximum ischemia time of six hours at hypothermia [1,2,3,4,5]. Final tissue damage is determined not only by the injury during preservation, but also by the tissue’s response to reperfusion (ischemia-reperfusion injury) [6,7]. This reperfusion response can activate a local or systemic inflammatory response that may exacerbate injury or produce injury in distant organs (e.g., lung oedema, kidney failure) [4]. Therefore, the evaluation of tissue damage following ischemia is only complete when the reperfusion phase is also assessed.

To date, cold storage is the gold standard in composite tissue preservation and early revascularisation remains the mainstay approach to minimise ischemic damage [8,9]. It is not always possible to reduce ischemia time to the 6 h maximum, for instance when vascular anastomoses are difficult and revisions are needed or in complex procedures such as vascularised composite allotransplantations (VCA, e.g., abdominal wall transplant) or limb replantation [10]. This raises the need for new techniques that can safely prolong composite tissue preservation [11]. Extracorporeal perfusion (ECP) found its origin in organ transplantation and slowly gained interest for preservation of composite tissues [12]. To date, ECP for composite tissue preservation is still experimental and studies that evaluated the reperfusion phase are scarce [13]. The longest described acellular ECP period followed by successful replantation is 24 h in porcine forelimbs [14].

In the current study, it is hypothesised that prolonged ECP is also possible in free flaps consisting almost entirely of skeletal muscle. Skeletal muscle is an important tissue to preserve in composite grafts since it is highly ischemia-sensitive and accounts for graft function [5,15,16]. Based on clinical appearance and gene expression patterns in previous experiments that assessed 36 h of ECP in musculocutaneous flaps, the maximum period of 18 h ECP was chosen. Outcomes after prolonged ECP were compared to a negative control group, consisting of flaps preserved by 4 h SCS [15,16].

## 2. Materials and Methods

Unilateral musculocutaneous free rectus abdominis flaps were raised from 15 Dutch Landrace pigs (mean weight 70.3 kg, range 62.9–83.5 kg). The contralateral side remained in situ, functioning as a control for flap appearance after replantation. Group allocation was randomised by using a random pattern generator at the start of the experiment. All animals received a bladder catheter and femoral arterial line for continuous monitoring of urine output and blood pressure. The use of animals was approved by the local and Dutch national animal experimentation committee (protocol-number 2016-0034-002) and followed the European Directive 2010/63/EU for the use and care of laboratory animals.

### 2.1. Surgical Procedure

All procedures were conducted under sterile conditions. Prophylactic antibiotics (amoxicillin 20 mg/kg) were administered intravenously before surgery. Pigs were heparinised up to 2 times the normal activated clotting time during surgery and to 1.5 times between procedures. All flaps were based on the superior epigastric artery and measured 12 × 9 cm. Directly after flap procurement, flaps were flushed with 150 cc heparin–saline solution (2 U/mL; room temperature) for a complete washout of blood. In the SCS group (*n* = 5), flaps were preserved for four hours, wrapped in dry gauzes in a sealed bag in ice water. In the ECP groups, flaps were perfused for 18 h. The abdominal wound was covered with moist gauzes and sterile, impermeable foil during ex vivo flap preservation. Flaps were replanted to their original vascular pedicle after preservation and were observed for another 12 h. After ending the experiment, pigs were euthanised with an overdose of Phenobarbital.

### 2.2. Perfusion System

**Figure 1:**
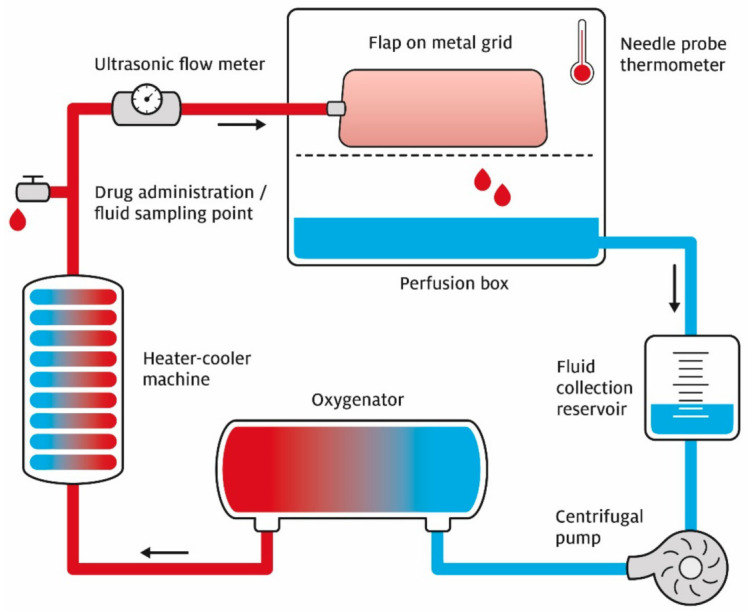
Schematic illustration of the extracorporeal perfusion set-up. The semi-closed perfusion set-up (closable box) with a flap in the perfusion box on top of a metal grid. This allows passive venous drainage of the preservation fluid into the collection reservoir. The centrifugal pump is pressure regulated at in-line ≤ 30 mmHg and the fluid is infused with a mix of 95% O_2_ and 5% CO_2_ in the oxygenator. The heater–cooler machine cools the fluid to 8–10 °C. There is a drug administration point and fluid sampling point. Before entering the flap via the artery, the ultrasonic flow meter measures flow per minute.

A semi-closed system was assembled for continuous, non-pulsatile extracorporeal perfusion with 1 L acellular preservation solution (Figure 1). Two solutions were compared: University of Wisconsin machine perfusion solution (UW-mp, *n* = 5) and Histidine–Tryptophan–Ketoglutarate (HTK, *n* = 5) [17]. Both solutions improved muscle preservation in several set-ups, including immersion and perfusion studies on muscle and solid organs [18,19].

Methylprednisolone (40 mg; Centrafarm B.V. Etten-Leur, The Netherlands) was added to the solution to prevent cellular swelling during perfusion. A centrifugal pump (BP-50 Bio-Pump^®^ Centrifugal Blood Pump, Medtronic, Fridley, MN, USA) was used to perfuse the tissue through an arterial catheter. The perfusion mode was pressure regulated, with a maximum in-line pressure of 30 mmHg. Venous outflow was passively collected in a reservoir before being filtered and pumped back into the system. A carbogen gas mixture of 95% O_2_ and 5% CO_2_ was continuously added to the preservation solution with a rate of 1 L/min by a hollow fibre oxygenator (Capiox RX05, Terumo, Shibuya, Tokio, Japan) [20]. This oxygen percentage was necessary for the fluid to reach an oxygenation of SO_2_ > 99% [21]. The solutions were cooled to 8–10 °C by a heater-cooler machine (HCU30, Maquet, Rastatt, Germany) according to the manufacturer’s recommendations. The observers were blinded for the used perfusion fluid (Table 1).

### 2.3. Baseline Measurements

Baseline samples of porcine arterial blood were taken from the femoral line before surgery and before pedicle transection. Samples were analysed for cytokine levels (IL-1β, IL-6, TNF-α using ELISA), for creatine kinase (CK) and for blood gas analysis including lactate using CG4+ iStat cartridges (Abbott, Princeton, NJ, USA). Flap weight was measured before preservation.

### 2.4. Measurements during Flap Preservation

Flap core temperature was measured using a needle probe thermometer. Perfusion solutions were neither refilled nor replenished, enabling measurements of flap metabolite accumulation in the solution (CK, lactate). Perfusion fluid samples were taken at 9 and 18 h of ECP to measure sO_2_ and flap metabolites. Flap weight was measured after preservation.

### 2.5. Measurements after Flap Replantation

Flap monitoring (skin colour, temperature and capillary refill) was performed hourly. In the case of abnormal controls, the pedicle was visually inspected and intervention took place if necessary. To visualise the perfusion of flaps, indocyanine-green (ICG) fluorescence angiography was used. ICG is a fluorescent dye that remains in the intravascular compartment after injection. The fluorescent signal can be visualised with a hand-held camera using near-infrared emission (PhotoDynamic Eye, Hamamatsu Photonics, Hamamatsu, Japan) [22]. A dose of 0.15 mg/kg was administered through the femoral artery line at 30 min, 4 h, 8 h and 12 h after replantation. The percentage of the fluorescent surface was recorded. Porcine arterial blood samples were taken at 1, 6 and 12 h after replantation for analysis of cytokines, CK and blood gas. Flaps were explanted after euthanizing the pigs, weight was recorded and flaps were fixed in buffered formaldehyde for histological sampling.

### 2.6. Histology

**Figure 2:**
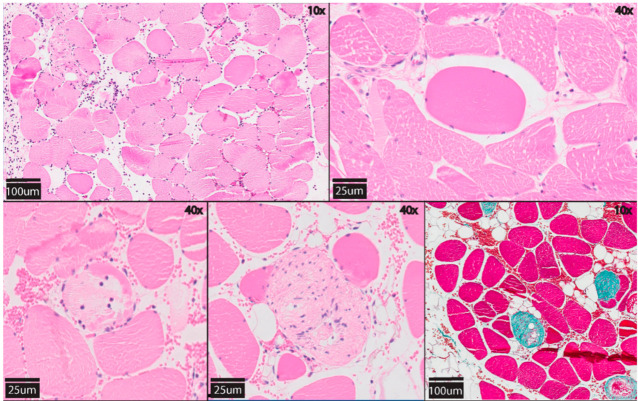
Morphological features of degenerative muscle fibres due to ischemia-reperfusion injury in transverse muscle sections stained with H&E at 12 h after flap replantation. Upper left: variation in muscle fibre shape and size. Oedema and inflammation are also seen. Upper right: hypoxic myocyte (rounded and hypereosinophilic). Lower left: neutrophil influx in the myocyte. Lower middle: necrotic, pale fibres and phagocytized myocytes. Lower right: Masson’s trichrome staining, fibrotic myocytes are stained green.

Transverse sections spanning the total flap width were cut at 3 µm and stained with haematoxylin and eosin (H&E) and Masson’s trichrome. All slides were evaluated by a blinded pathologist with light microscopy in 10 randomly selected high-power fields using ×20 magnification. A self-developed and simplified version of the ‘Histologic injury severity score’ as presented by Muller et al. was used for histological scoring since no specific score is available [23]. Four subgroups of histologic alterations that are indicators for muscle damage were scored on a scale from 0 (no damage) to 3 points (severe damage). These subgroups are: (1) damaged muscle fibres (=hypoxic fibres + necrosis + phagocytic cells), (2) inflammation, (3) interstitial oedema and (4) variation in shape and size of myocytes (Figure 2). The sum of all categories resulted in a total score between 0 and 12 points (Table 2) [1].

### 2.7. Statistical Analysis

The differences in distribution between groups were analysed with Kruskal–Wallis tests for single-measurement parameters. Parameters with repeated measures were analysed using a mixed model analysis (e.g., weight, CK, lactate). Analyses were performed in SAS 9.4 (SAS Institute Inc, Cary, NC, USA) and *p*-values of ≤0.05 were considered statistically significant.

## 3. Results

Mean pig weight, flush duration and replantation time were comparable between groups (Table 3). Mean off-pedicle preservation time was prolonged with >13.5 h in ECP groups.

### 3.1. Measurements during Flap Preservation

The core temperature in the SCS-preserved flaps dropped to ≤8 °C within 3 h of SCS. Flap core temperature during UW-mp perfusion was significantly higher at all times compared to HTK perfusion (mean 19.4 °C, SD 2.1 vs. 16.8 °C, SD 0.8; *p* = 0.027). The mean perfusion pressure was 26 mmHg during ECP with HTK and 30 mmHg during ECP with UW solution. The mean sO_2_ was >99% in influx perfusion solution and 30–40% in efflux solution. These values approximate physiological circumstances. The accumulation of CK and lactate in the preservation solutions during ECP was comparable between groups.

### 3.2. Measurements after Flap Replantation

**Figure 3:**
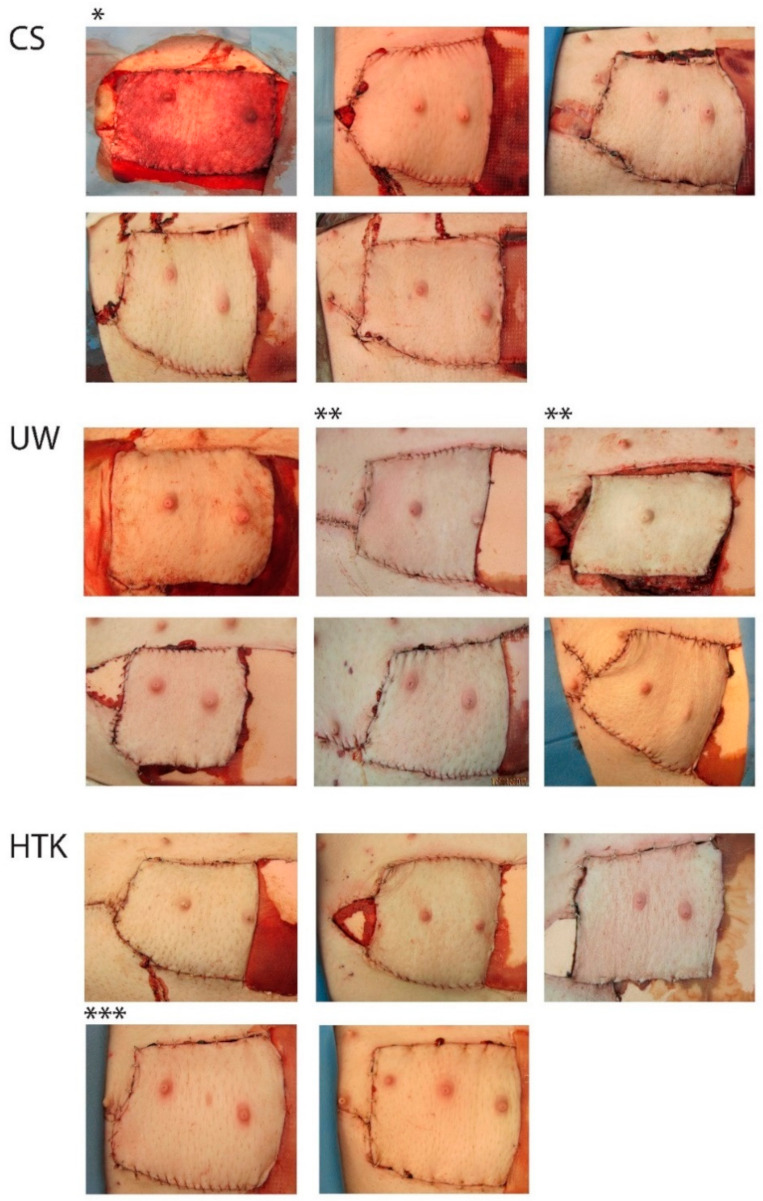
Photographs of all flaps at 12 h after replantation. * Flap 1 was excluded due to therapy-resistant venous congestion and later arterial obstruction. ** Flap 5 at 11 h (left) and 12 h (right) after flap replantation (before and after acute arterial thrombosis). *** Flap 14 underwent successful revision of the arterial pedicle at 8 h after replantation and had normal appearance afterwards. Abbreviations: CS; cold storage, UW; University of Wisconsin solution, HTK; histidine-tryptophan-ketoglutarate solution.

Flap #1 (SCS group) developed venous congestion directly after replantation that did not clear after revision nor anastomosis of a second vein. The flap additionally showed a decreased and later absent arterial inflow on ICG angiography. Since there was no obvious explanation for this problem (e.g., pedicle thrombosis), the flap was excluded from the study, leaving four flaps in the SCS group (Figure 3).

Two flaps from the ECP groups developed acute arterial thrombosis after replantation. This developed for Flap #14 (HTK-group) at eight hours after replantation, for which successful revision surgery was performed without further problems. Flap #5 (UW group) developed arterial thrombosis at 11.8 h after replantation. Since this event was near the end of the experiment, no revision was undertaken (Figure 3). All other flaps had uneventful follow-ups, with complete and homogenous perfusion patterns on ICG angiography.

### 3.3. Laboratory Measurements

**Figure 4:**
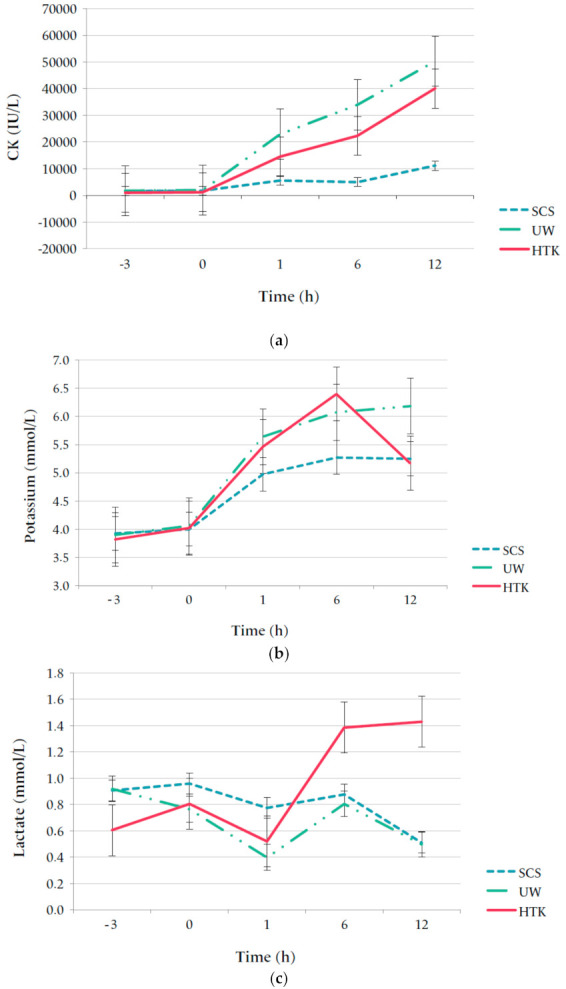
Mean CK (**a**), potassium (**b**) and lactate (**c**) per pig. Arterial measurements were taken during flap retrieval and after flap replantation. Porcine reference values: CK 153–5427 U/L, potassium 3.7–6.1 mmol/L, lactate unknown [24].

Systemic cytokine levels were low or undetectable in all flaps. Porcine arterial CK levels increased over time in all groups after flap replantation but were significantly lower in the SCS group compared to the ECP groups (mixed model analysis, *p* < 0.001). A comparison between the ECP groups showed a trend towards lower CK in the HTK group at six hours after replantation (*p* = 0.051) but comparable CK levels at 12 h (*p* = 0.095). The arterial potassium after replantation were comparable between all groups (*p* = 0.529). Lactate values showed an increase in the HTK group from 6 hours after replantation (*p* = 0.522, Figure 4).

### 3.4. Histology

**Figure 5:**
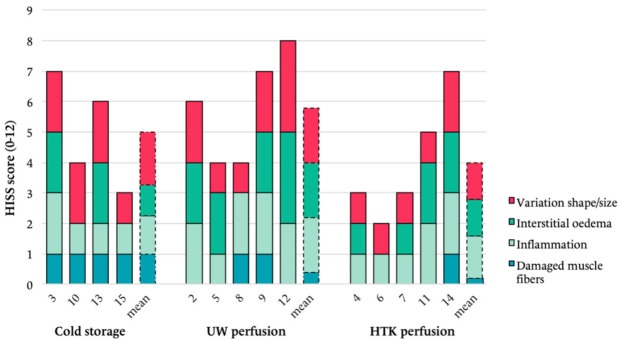
Boxplot showing an overview of histological changes at the study endpoint indicating ischemia-reperfusion injury per flap and group. Cell count per 10 high-power fields at 20× magnification. Scoring for inflammation and interstitial oedema: 0 = no, 1 = minimal, 2 = intermediate, 3 = diffuse.

The mean score of ischemia-induced alternations (HISS) was 5.0 (SD 1.8) in SCS, 5.6 (SD 1.9) in UW and 4.0 (SD 2.0) in HTK-preserved flaps (Kruskal–Wallis, *p* = 0.366). There were small differences in sub-scores between the preservation groups, without reaching statistical significance (Figure 5). The number of damaged muscle fibres was lowest in the HTK group (mean 0.2, SD 0.4), followed by the UW group (mean 0.6, SD 0.5) and SCS group (mean 1.0, SD 0.0; *p* = 0.214).

### 3.5. Flap Weight

**Figure 6:**
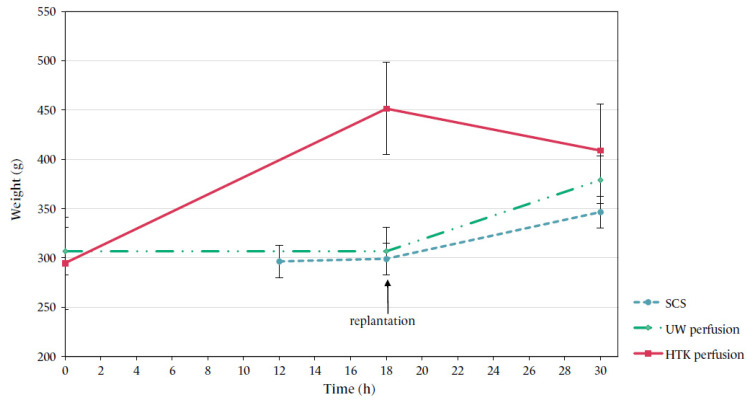
Mean flap weight over time per group. Time points of flap weight measurements: directly after flap retrieval, after 4 h cold storage or 18 h ECP and after 12 h reperfusion.

In contrast to UW-mp-perfused flaps, HTK-perfused flaps showed a visible weight increase during ECP (HTK: +156.5 g (53%); UW: −0.22 g (0%), mixed model analysis *p* ≤ 0.001). After replantation, SCS and UW-mp flaps showed a moderate weight increase (SCS: +47.3 g (17%), UW: +66.8 g (24%), *p* = 0.674), but the weight of HTK-preserved flaps decreased, with a mean of 72.6 g (−27%). When comparing the final flap weight, there was a statistically significant difference between HTK and SCS (*p* = 0.008), but not between the two ECP groups (*p* = 0.169, Figure 6).

## 4. Discussion

Tissue preservation by the current standard of SCS is limited by the ischemic tolerance per tissue type [4]. Since short ischemia time cannot always be guaranteed, there is a need to explore techniques that can safely prolong tissue preservation. This study assessed the early reperfusion phase after 18 h porcine rectus abdominus flap preservation using oxygenated mid-thermic ECP with acellular fluids. Histology and clinical appearance were found to be comparable to 4 h SCS.

Interestingly, upon histological evaluation, muscle damage after 18 h ECP and replantation seemed to be comparable to 4 h SCS and replantation (Figure 5). These findings confirm that flap histology is well preserved in the early replantation phase following prolonged ECP with retained muscle architecture. The limb preservation study by Kueckelhaus et al. described similar results after acellular perfusion and even suggested superiority of 12 h ECP with Perfadex over 4 h SCS [25]. Of important note is that ischemia-reperfusion injury histologically presents as a heterogenic phenomenon, which therefore is prone to sampling error [4]. To minimise this, complete flaps were fixed in formaldehyde and cross-sections spanning the total flap width were taken for histological scoring.

CK is an enzyme that is present in cells that rapidly consume ATP (e.g., skeletal or cardiac muscle). In response to muscle activity or damage, CK leaks into blood, leading to increased serum levels [26]. CK levels in this experiment were higher after ECP than SCS, suggesting more muscle damage and inflammation. However, in this field of research, CK levels remain to be related to the extent of muscle damage. Although a total increase in CK to 40,000–50,000 IE/L is high, other articles which presented good tissue preservation described similar levels of increase. Fahradyan et al. described an increase in CK to 64,333 U/L after 24 h of blood-based perfusion in porcine limbs [27]. Duraes and colleagues found mean CK levels to be >27,000 U/L after 12 h normothermic blood-based porcine limb perfusion, while muscle contraction was 100% preserved [28]. A factor that could have possibly contributed to increased CK levels in the ECP groups was the longer anesthesia time, especially since pigs have the tendency to develop muscle spasms during anesthesia. Nevertheless, comparing CK levels between ECP groups showed a trend towards lower values after HTK perfusion, in line with the lower histological scores in this group. The relation between CK and muscle damage remains unclear and warrants further research in this field of medicine, specifically focussing on finding a cut-off value for clinically relevant muscle damage.

Müller et al. measured systemic cytokine levels to assess the immune response after porcine limb replantation [23]. However, we could not reproduce these measurements. Systemic cytokine levels after flap replantation remained very low or undetectable in all groups, which can be interpreted as a failure to detect cytokines though dilution in the systemic circulation, but also as the absence of a systemic immune response, indicating a low degree of ischemic injury. For future research purposes, sensitivity would increase if local cytokines levels could be measured.

The choice between UW-mp and HTK solution for composite tissue ECP remains a topic of debate [12]. In this experiment, HTK perfusion seemed to result in slightly better histological outcomes, while UW-mp perfusion resulted in lower weight increases during perfusion. The development of oedema is very common in tissue reperfusion and fairly difficult to circumvent. While oedema will mostly disappear in the first days after reperfusion, extreme oedema should be prevented as it may compromise cellular microcirculation. The reported weight gain after acellular perfusion is variable, with rates between 10 and 84% after 6–12 h perfusion [29,30,31]. This large range can be explained by the heterogeneity of perfusion fluids. In this experiment, UW-mp perfusion resulted in a stable weight. This could be explained by the high viscosity of this solution due to the addition of three oncotic agents: hydroxyethyl starch (colloid), gluconate and raffinose (impermeants) [32]. HTK solution contains no colloids, but only mannitol (impermeant) as an oncotic agent. Interestingly, after weight increase during perfusion, HTK flaps responded favourably to replantation in terms of oedema and showed a reduction in mean weight, while the SCS and UW-mp flaps increased in weight in this period [33]. Given the promising results of HTK in this study, future research could explore the possibility of a modified HTK solution aiming to reduce weight gain during perfusion, for instance by adding a plasma expander to the fluid.

Even though perfusate temperature was kept below 8 °C, gradual rewarming of the solution after passing though the heater-cooler element before entering the flap unintentionally resulted in higher flap core temperatures. Since tissue temperature is closely related to oxygen consumption and ischemia rate, this could have negatively influenced results [9]. In future research, the perfusion set-up could be improved to prevent rewarming of the preservation solutions.

The flap failure rate of 6.7% (1/15) is in line with the rate of approximately 5% in human research, considering the higher thrombogenic potential of pigs compared to humans [34,35,36]. Furthermore, previous research has learned that muscle flaps provide a very sensitive model for failure [37]. Two ECP flaps developed acute arterial occlusion in this experiment. Perfusion damage to the endothelial layer was assessed as a possible causing factor, but could not be confirmed on histology. To our knowledge, there is one other study that presents some information on graft failure after ECP in pigs. This study by Muller et al. had a complication rate of 32% after 12 h ECP and replantation of porcine limbs. Infection and thrombosis were both scored as a complication but not further elucidated [23].

A disadvantage of this study is the short follow-up after replantation. This article therefore only focussed on the acute phase after ECP. The twelve-hour follow-up period after replantation was a pragmatic choice, supported by the fact that in free flap replantation, 80% of flap compromise occurs within 24 h after surgery, of which 80% of the cases present within the first 12 h [38]. Following evidence indicating that histological findings at 3 h post-transplantation already correlate with the findings at one week after transplantation, the results from this research might serve as a guide for follow-up experiments [25]. Due to strict Dutch regulations in animal welfare, an animal survival study with longer follow-up was not found to be an ethically approvable option based on currently available evidence, and therefore remains a future goal. An advantage of this study was the use of a large animal model and very ischemia-sensitive tissue, enhancing the translational value to a human population. Furthermore, flap viability was evaluated during both perfusion and replantation, enabling the assessment of the early ischemia-reperfusion reaction. The measuring of muscle function and its relation to histology remains a future goal that can help further increase the understanding of muscle tissue response to prolonged extracorporeal muscle perfusion.

## 5. Conclusions

Prolonging preservation time from 4 to 18 h with acellular ECP seems feasible in highly ischemia-sensitive muscle flaps. This is a three-fold elongation of the current maximum allowable muscle ischemia.

## Figures and Tables

**Table 1:** Composition of UW-mp and HTK solution.

Component	UW-mp	HTK
Osmolality (mOsm/L)	320	310
Potassium (mmol/L)	25	9
Sodium (mmol/L)	80	15
Calcium (mmol/L)	0.5	0.015
Scavangers	Glutathione, allopurinol	Tryptophan, mannitol
Buffers	Phosphate	Histidine
Energy delivery	Adenosine, phosphate	Ketoglutarate
Impermeants	HES, gluconate, raffinose	Mannitol

Abbreviations: UW-mp—University of Wisconsin machine perfusion solution; HTK—Histidine–Tryptophan–Ketoglutarate.

**Table 2:** Proposed histologic scoring system for hypoxia-induced muscle tissue injury in extracorporeal perfusion settings.

Morphological Changes	Categories
Interstitial oedema	0. No significant increase 1. Minimal 2. Intermediate 3. Severe/diffuse
Inflammation	0. Not significant 1. Minimal 2. Intermediate 3. Diffuse
Variation in shape and size of myocytes	0. Homogeneous 1. Mild heterogeneous 2. Intermediate heterogenous 3. Severe heterogenous
Damaged muscle fibres *	0. 0–5 myocytes 1. 6–20 myocytes 2. 21–50 myocytes 3. >51 myocytes

* Sum of damaged fibers at 10 high-power fields at 20× magnification; total score 0–12, a higher score equals more muscle damage. The scoring system is based on findings of Scully and Hughes [1], and the histologic ischemia severity score (HISS) by Muller et al. [23].

**Table 3:** Characteristics of the three preservation groups presenting means, standard deviation and *p*-values.

	I. Cold Storage	II. UW-mp Perfusion	III. HTK Perfusion	*p*-Value
Pig weight (kg)	72 ± 8	67 ± 4	71 ± 6	0.946
Flush duration (min)	18 ± 6	22 ± 8	18 ± 7	0.738
Replantation time (min)	73 ± 18	59 ± 25	58 ± 25	0.201
Total off-pedicle time (h)	5 ± 0.3	19 ± 0.8	19 ± 0.5	0.009 *

The flap from pig 1 in the cold storage group was excluded due to technical problems directly after replantation. * Kruskal–Wallis analysis, *p* ≤ 0.05 is considered statistically significant. Abbreviations: UW-mp—University of Wisconsin machine perfusion solution; HTK—Histidine–Tryptophan–Ketoglutarate solution.

## Data Availability

Data available on request due to ethical policy. The data presented in this study are available on request from the corresponding author.

## References

[1] Scully R.E., Hughes C.W. (1956). The pathology of ischemia of skeletal muscle in man; a description of early changes in muscles of the extremities following damage to major peripheral arteries on the battlefield. Am. J. Pathol..

[2] Harman J.W. (1947). A histologic study of skeletal muscle in acute ischemia. Am. J. Pathol..

[3] Datta N., Devaney S.G., Busuttil R.W., Azari K., Kupiec-Weglinski J.W. (2017). Prolonged Cold Ischemia Time Results in Local and Remote Organ Dysfunction in a Murine Model of Vascularized Composite Transplantation. Arab. Archaeol. Epigr..

[4] Blaisdell F. (2002). The pathophysiology of skeletal muscle ischemia and the reperfusion syndrome: A review. Cardiovasc. Surg..

[5] Eckert P., Schnackerz K. (1991). Ischemic Tolerance of Human Skeletal Muscle. Ann. Plast. Surg..

[6] Kalogeris T., Baines C.P., Krenz M., Korthuis R.J. (2012). Cell Biology of Ischemia/Reperfusion Injury. Int. Rev. Cell Mol. Biol..

[7] Sternbergh W.C., Adelman B. (1992). The temporal relationship between endothelial cell dysfunction and skeletal muscle damage after ischemia and reperfusion. J. Vasc. Surg..

[8] Amin K.R., Ball A.L., Chhina C., Edge R.J., Stone J.P., Critchley W.R., Wong J.K., Fildes J.E. (2018). Ex-vivo flush of the limb allograft reduces inflammatory burden prior to transplantation. J. Plast. Reconstr. Aesthetic Surg..

[9] Dickey R.M., Hembd A.S., Fruge S., Haddock N., Papas K.K., Suszynski T.M. (2020). Composite Tissue Preservation. Ann. Plast. Surg..

[10] Barnes J., Issa F., Vrakas G., Friend P., Giele H. (2016). The abdominal wall transplant as a sentinel skin graft. Curr. Opin. Organ Transplant..

[11] Amin K.R., Wong J.K., Fildes J.E. (2017). Strategies to Reduce Ischemia Reperfusion Injury in Vascularized Composite Allotransplantation of the Limb. J. Hand Surg..

[12] Latchana N. (2015). Preservation solutions used during abdominal transplantation: Current status and outcomes. World J. Transplant..

[13] Kruit A.S., Winters H., Van Luijk J., Schreinemachers M.-C.J., Ulrich D.J. (2018). Current insights into extracorporeal perfusion of free tissue flaps and extremities: A systematic review and data synthesis. J. Surg. Res..

[14] Krezdorn N., Macleod F., Tasigiorgos S., Turk M., Wo L., Kiwanuka H., Lopdrup B.R., Kollar B., Edelman E.R., Pomahac B. (2019). Twenty-Four–Hour Ex Vivo Perfusion with Acellular Solution Enables Successful Replantation of Porcine Forelimbs. Plast. Reconstr. Surg..

[15] Kruit A.S., Schreinemachers M.-C.J., Koers E.J., Zegers H.J., Hummelink S., Ulrich D.J. (2019). Successful Long-term Extracorporeal Perfusion of Free Musculocutaneous Flaps in a Porcine Model. J. Surg. Res..

[16] Kruit A.S., Smits L., Pouwels A., Schreinemachers M.-C.J., Hummelink S.L., Ulrich D.J. (2019). Ex-vivo perfusion as a successful strategy for reduction of ischemia-reperfusion injury in prolonged muscle flap preservation—A gene expression study. Gene.

[17] Karangwa S.A., Dutkowski P., Fontes P., Friend P.J., Guarrera J.V., Markmann J.F., Mergental H., Minor T., Quintini C., Selzner M. (2016). Machine Perfusion of Donor Livers for Transplantation: A Proposal for Standardized Nomenclature and Reporting Guidelines. Arab. Archaeol. Epigr..

[18] Kaltenborn A., Gwiasda J., Amelung V., Krauth C., Lehner F., Braun F., Klempnauer J., Reichert B., Schrem H. (2014). Comparable outcome of liver transplantation with Histidine-Tryptophan-Ketoglutarate vs. University of Wisconsin preservation solution: A retrospective observational double-center trial. BMC Gastroenterol..

[19] Van der Heijden E.P., Kroese A.B., Werker P.M., Grabietz P.D., de Jong M.B., Bär P.R., Kon M. (1998). Function of rat skeletal muscles after storage at 10 degrees C in various preservation solutions. Clin Sci..

[20] Fuller B.J., Lee C.Y. (2007). Hypothermic perfusion preservation: The future of organ preservation revisited?. Cryobiology.

[21] Ishikawa S., Ueda K., Neya K., Abe K., Kugawa S., Kawasaki A., Nishizawa S., Yamaoka K. (2010). Effects of original crystalloid cardioplegia followed by additional blood cardioplegia: Treatments for prolonged cardiac arrest. Ann. Thorac. Cardiovasc. Surg..

[22] Munabi N.C., Olorunnipa O.B., Goltsman D., Rohde C.H., Ascherman J.A. (2014). The ability of intra-operative perfusion mapping with laser-assisted indocyanine green angiography to predict mastectomy flap necrosis in breast reconstruction: A prospective trial. J. Plast. Reconstr. Aesthetic Surg..

[23] Müller S., Constantinescu M.A., Kiermeir D.M., Gajanayake T., Bongoni A.K., Vollbach F.H., Meoli M., Plock J., Jenni H., Banic A. (2013). Ischemia/reperfusion injury of porcine limbs after extracorporeal perfusion. J. Surg. Res..

[24] Cooper C.A., Moraes L.E., Murray J.D., Owens S.D. (2014). Hematologic and biochemical reference intervals for specific pathogen free 6-week-old Hampshire-Yorkshire crossbred pigs. J. Anim. Sci. Biotechnol..

[25] Kueckelhaus M., Dermietzel A., Alhefzi M., Aycart M.A., Fischer S., Krezdorn N., Wo L., Maarouf O.H., Riella L.V., Abdi R. (2017). Acellular Hypothermic Extracorporeal Perfusion Extends Allowable Ischemia Time in a Porcine Whole Limb Replantation Model. Plast. Reconstr. Surg..

[26] Venance S.L. (2016). Approach to the Patient With HyperCKemia. Contin. Lifelong Learn. Neurol..

[27] Fahradyan V., Said S.A., Ordenana C., Pozza E.D., Frautschi R., Duraes E.F.R., Madajka-Niemeyer M., Papay F.A., Rampazzo A., Gharb B.B. (2020). Extended ex vivo normothermic perfusion for preservation of vascularized composite allografts. Artif. Organs.

[28] Duraes E.F.R., Madajka M., Frautschi R., Soliman B., Cakmakoglu C., Barnett A., Tadisina K., Liu Q., Grady P., Quintini C. (2017). Developing a protocol for normothermic ex-situ limb perfusion. Microsurgery.

[29] Araki J., Sakai H., Takeuchi D., Kagaya Y., Tashiro K., Naito M., Mihara M., Narushima M., Iida T., Koshima I. (2015). Normothermic Preservation of the Rat Hind Limb With Artificial Oxygen-carrying Hemoglobin Vesicles. Transplantation.

[30] Kueckelhaus M., Fischer S., Sisk G., Kiwanuka H., Bueno E.M., Dermietzel A., Alhefzi M., Aycart M., Diehm Y., Pomahac B. (2016). A Mobile Extracorporeal Extremity Salvage System for Replantation and Transplantation. Ann. Plast. Surg..

[31] Taeger C.D., Friedrich O., Drechsler C., Weigand A., Hobe F., Geppert C.I., Münch F., Birkholz T., Buchholz R., Horch R.E. (2016). Hydroxyethyl starch solution for extracorporeal tissue perfusion. Clin. Hemorheol. Microcirc..

[32] Jaeschke H. (1996). Preservation injury: Mechanisms, prevention and consequences. J. Hepatol..

[33] Homer-Vanniasinkam S., Rowlands T., Hardy S., Gough M. (2001). Skeletal Muscle Ischaemia-reperfusion Injury: Further Characterisation of a Rodent Model. Eur. J. Vasc. Endovasc. Surg..

[34] Yu P., Chang D.W., Miller M.J., Reece G., Robb G.L. (2009). Analysis of 49 cases of flap compromise in 1310 free flaps for head and neck reconstruction. Head Neck.

[35] Zoccali G., Molina A., Farhadi J. (2017). Is long-term post-operative monitoring of microsurgical flaps still necessary?. J. Plast. Reconstr. Aesthetic Surg..

[36] Bigdeli A.K., Gazyakan E., Schmidt V.J., Bauer C., Germann G., Radu C.A., Kneser U., Hirche C. (2018). Long-Term Outcome after Successful Lower Extremity Free Flap Salvage. J. Reconstr. Microsurg..

[37] Slater N.J., Zegers H.J., Küsters B., Beune T., van Swieten H.A., Ulrich D.J. (2016). Ex-vivo oxygenated perfusion of free flaps during ischemia time: A feasibility study in a porcine model and preliminary results. J. Surg. Res..

[38] Chen K.-T., Mardini S., Chuang D.C.-C., Lin C.-H., Cheng M.-H., Lin Y.-T., Huang W.-C., Tsao C.-K., Wei F.-C. (2007). Timing of Presentation of the First Signs of Vascular Compromise Dictates the Salvage Outcome of Free Flap Transfers. Plast. Reconstr. Surg..

